# Retrograde Intubation in Advanced Nasopharyngeal Carcinoma With Airway Obstruction: A Case Report and Literature Review

**DOI:** 10.1002/ccr3.70436

**Published:** 2025-04-16

**Authors:** Laxman Wagle, Dhiraj Raj Regmi, Sangharsha Thapa, Mustafa Abdulmahdi

**Affiliations:** ^1^ Department of Internal Medicine Ascension Saint Agnes Hospital Baltimore Maryland USA; ^2^ Department of Internal Medicine Residency MedStar Health Baltimore Maryland USA; ^3^ Department of Neurology Westchester Medical Center Valhalla New York USA

**Keywords:** airway obstruction, difficult airway management, endotracheal intubation, head and neck cancer, nasopharyngeal carcinoma, retrograde intubation

## Abstract

Retrograde intubation is a valuable technique for securing the airway in patients with advanced nasopharyngeal carcinoma causing airway obstruction. In this case, retrograde intubation enabled successful airway management when conventional methods failed, highlighting its role in complex head and neck cancer cases. It offers low complication risks and high success rates.

## 1 Introduction

Retrograde intubation is a surgical airway management technique involving translaryngeal guidance for orotracheal or nasotracheal intubation [1]. This approach is often critical when conventional intubation methods are ineffective, such as in cases of airway obstruction due to large head and neck tumors. For patients with compromised airways, management typically begins with flexible nasoendoscopy to assess the degree of obstruction, followed by endotracheal intubation. When standard intubation techniques fail, procedures such as tracheostomy and rigid bronchoscopy may be considered, while temporary airway support may be achieved through a transtracheal cannula or cricothyroidotomy with jet ventilation [2]. Airway management for head and neck tumors, however, presents unique challenges that require specialized consideration [3].

This report describes the case of a 36‐year‐old male with nasopharyngeal carcinoma, who presented with severe airway obstruction. When standard intubation techniques were unsuccessful due to the tumor burden, retrograde intubation allowed for secure airway access and enabled further therapeutic interventions. This case highlights the value of retrograde intubation in managing difficult airways, particularly for patients with complex head and neck pathology, and emphasizes its relatively high success rate and low complication profile even in emergency settings.

## 2 Case History/Examination

A 36‐year‐old African American male with stage IVA Epstein–Barr virus‐positive squamous cell carcinoma of the nasopharynx, diagnosed 8 months prior, presented with worsening dyspnea, dysphagia, and difficulty managing secretions. He was actively receiving chemotherapy with cisplatin and gemcitabine. His symptoms intensified following his most recent chemotherapy cycle, completed the previous day.

Upon arrival, the patient's vital signs indicated significant respiratory distress with a respiratory rate of 30 breaths per minute and oxygen saturation of 98% on room air. Physical examination revealed a muffled voice, drooling, and the use of accessory muscles for respiration. Inspiratory and expiratory stridor were also noted.

## 3 Differential Diagnosis

We thought the patient's symptoms could be due to

1. Airway obstruction due to advanced nasopharyngeal carcinoma.
2. Acute airway compromise secondary to chemotherapy‐induced mucositis or infection.
3. Aspiration pneumonia.
4. Acute respiratory distress syndrome.

**FIGURE 1:**
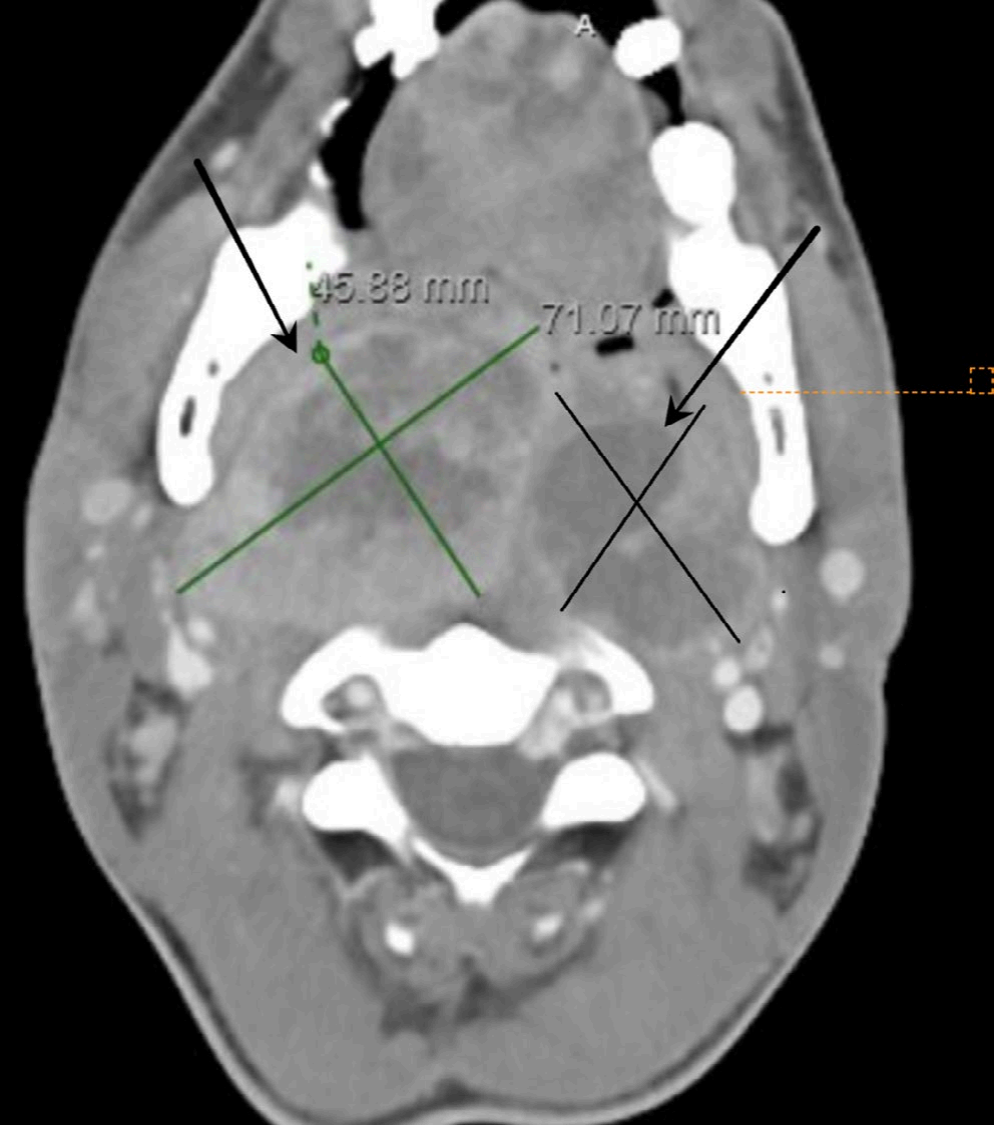
CT scan showing right (green cross) and left nasopharyngeal mass (black cross) due to advanced nasopharyngeal carcinoma.

**FIGURE 2:**
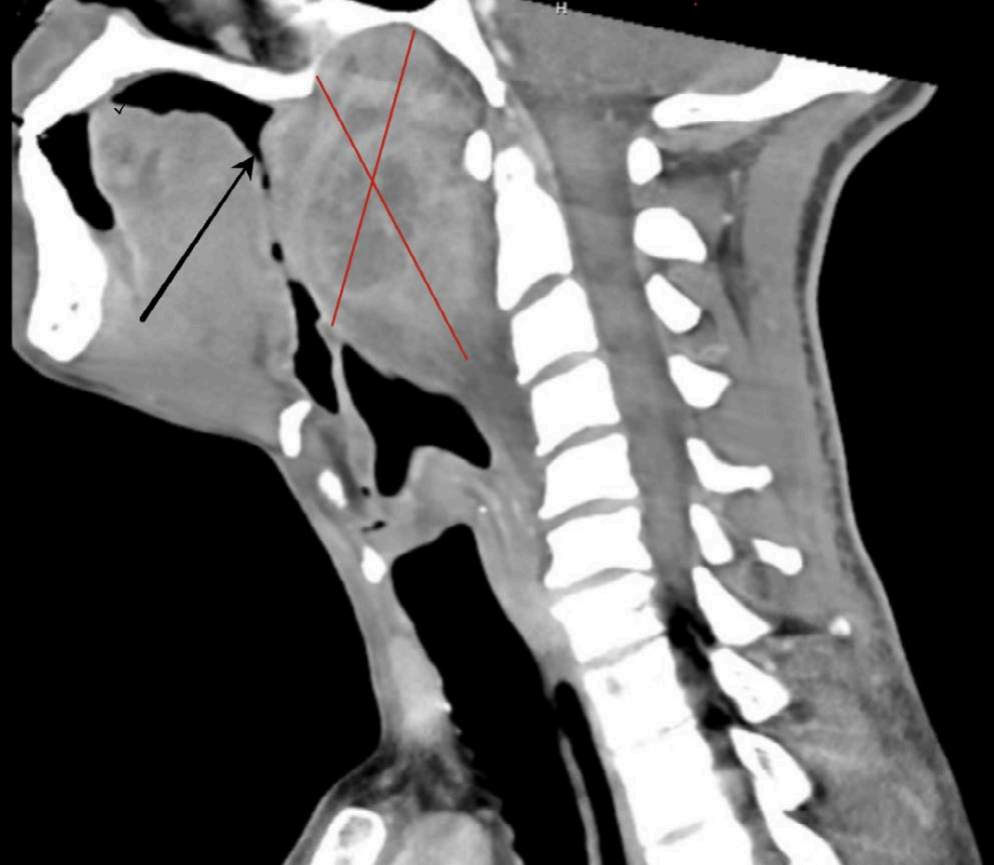
CT scan showing nasopharyngeal carcinoma (red cross) and near complete obstruction of posterior airways (arrow).

We performed an extensive workup, including a computed tomography (CT) scan, which revealed a large, necrotic mass causing near‐total obstruction of the posterior pharynx (Figures 1 and 2). Laboratory analysis showed neutrophil‐predominant leukocytosis with respiratory acidosis, indicating significant respiratory compromise.

## 4 Conclusion and Results (Outcome and Follow‐Up)

While the patient's airway obstruction was primarily due to the large tumor mass, it is important to consider the other potential causes of acute respiratory distress, such as infections or treatment‐related complications. While awake tracheostomy is often considered a definitive solution for anticipated difficult airways in head and neck cancer patients, several factors influenced our initial decision against this approach. The patient's nasopharyngeal tumor demonstrated significant cervical extension, resulting in distorted neck anatomy that obscured typical surgical landmarks. Furthermore, the tumor's hypervascularity posed a substantial risk of catastrophic bleeding during the procedure. The patient's severe respiratory distress complicated matters further, as maintaining optimal positioning for a surgical airway would have been challenging and potentially dangerous. These factors, combined with the acute nature of the presentation, led us to opt for retrograde intubation as a bridge to a more controlled, elective tracheostomy once the airway was secured. Awake fiberoptic intubation was attempted but abandoned due to copious secretions and bleeding obscuring the view, significant tumor‐related anatomical distortion, and the patient's inability to maintain position due to respiratory distress. Video laryngoscopy was attempted under topical anesthesia with minimal sedation but failed due to the inability to visualize landmarks because of the tumor mass, limited mouth opening, and the presence of secretions. Due to the failure of video laryngoscopy and the inability to visualize the airway because of tumor obstruction, retrograde intubation was deemed necessary.

After preoxygenation, the patient received light sedation with intravenous ketamine. Local anesthesia was administered at the cricothyroid membrane, and an angiocatheter was inserted into the anterior tracheal wall to guide a wire retrograde through the oral cavity. A 6.0 endotracheal tube (ETT) was advanced over the wire, with confirmation of placement via end‐tidal CO_2_ and bronchoscopy. The risk of bleeding during guidewire insertion was carefully considered, given its potential to exacerbate airway obstruction. Preparatory measures included verification of coagulation status and implementation of a comprehensive backup strategy. An ENT surgical team remained on immediate standby, with emergency tracheostomy equipment readily available. The procedure prioritized controlled guidewire manipulation while maintaining preparedness for urgent surgical airway intervention if required. The patient maintained satisfactory oxygen saturation throughout the procedure. After successful intubation, a bronchoscopy‐guided percutaneous tracheostomy was performed, and the ETT was removed. The patient was subsequently treated with steroids and palliative radiation therapy. Following clinical stabilization, he was discharged with close outpatient follow‐up.

## 5 Discussion

Retrograde endotracheal intubation (REI) was first introduced by Butler and Cirillo in 1960 as a method to facilitate airway access in patients where conventional endotracheal intubation approaches were unfeasible due to anatomical abnormalities or obstructive pathologies [4, 5]. The classic REI technique involves the percutaneous puncture of the cricothyroid membrane, insertion of a wire, and subsequent placement of an ETT over the guide wire to secure the airway [4, 6]. Over time, multiple variations of this technique have been developed, making REI a valuable, versatile tool in the arsenal of emergency airway management, particularly in complex cases involving head and neck malignancies where other techniques may be less effective or entirely unsuccessful [5, 7].

### 5.1 Challenges of Conventional Intubation Techniques

In the context of head and neck cancer, such as advanced nasopharyngeal carcinoma, traditional airway management methods, including video laryngoscopy and fiberoptic intubation, often prove insufficient. Tumor masses in these patients can cause extensive anatomical distortion, obscuring the view of the vocal cords and preventing the passage of standard intubation equipment [7]. As such, the presence of extensive head and neck pathology necessitates alternative approaches. Although fiberoptic intubation is widely regarded as the gold standard for difficult airways, it can be ineffective or impractical in emergency settings, particularly if there is copious bleeding, secretion, or direct tumor invasion, all of which can obstruct the fiberoptic view [7, 8].

### 5.2 Role of Retrograde Intubation in Complex Airway Management

REI offers a reliable solution when conventional intubation techniques have failed or are contraindicated. Unlike tracheostomy, which carries considerable morbidity including hemorrhage, infection, tube displacement, and a small but notable risk of mortality, REI is minimally invasive and carries a comparatively lower complication profile [4, 6]. The most common adverse effects associated with REI, such as sore throat, minor bleeding, and subcutaneous emphysema, are generally self‐limiting and do not typically necessitate additional interventions [6]. By puncturing the cricothyroid membrane and using a guidewire to facilitate intubation, clinicians can bypass the obstructed or distorted upper airway anatomy and gain immediate access to the trachea, thus ensuring oxygenation and ventilation in time‐sensitive situations [4, 5].

### 5.3 Comparative Efficacy and Success Rates of Retrograde Intubation

Retrospective analyses have shown that, in cases of head and neck cancer, fiberoptic intubation remains the preferred method for airway management, with nasal intubation being employed in 92.4% of cases and oral intubation in 7% [7]. Emergency tracheostomies, by contrast, are performed only in 0.6% of cases, reflecting a general preference for less invasive approaches whenever possible [7]. However, both nasal and fiberoptic intubation carry inherent risks in complex cases, including nasal bleeding, retropharyngeal injury, and nosocomial sinusitis, which can complicate the patient's clinical course and prolong recovery [8]. In this light, REI emerges as a safer, more controlled alternative that reduces trauma to surrounding structures and provides effective airway access with minimal interference from the upper airway's pathological features [4, 6].

### 5.4 Advantages of Retrograde Intubation in Emergency Settings

The success and relative simplicity of REI makes it an essential skill for emergency and critical care providers, especially in settings where specialized fiber optic equipment may not be readily available. In a study by Weksler et al., retrograde intubation was demonstrated as a valuable alternative when fiberoptic intubation was either unavailable or ineffective, further supporting its role as a vital technique in emergency airway management [8]. REI can be performed under local anesthesia and does not require advanced visualization tools, which enhances its applicability in a wide range of clinical scenarios, from resource‐limited environments to complex emergency cases in tertiary care facilities [4, 6].

### 5.5 Training and Clinical Proficiency in Retrograde Intubation

Given its value as an emergency airway management tool, REI should be a standard component of training for clinicians managing patients with head and neck malignancies, as well as those working in high‐acuity settings. Familiarity with REI can significantly improve outcomes by reducing the risks associated with delayed or failed airway access [4, 6]. Hospitals, emergency departments, and emergency medical services (EMS) should incorporate REI training into their airway management protocols, especially for staff frequently managing airway obstructions due to complex pathologies [5, 9]. According to recent recommendations, proficiency in REI enhances clinician confidence in managing airways in patients with unique anatomical challenges and optimizes patient safety in high‐risk cases.

## Author Contributions

**Laxman Wagle:** conceptualization, data curation, formal analysis, methodology, supervision, validation, visualization, writing – original draft, writing – review and editing. **Dhiraj Raj Regmi:** conceptualization, formal analysis, methodology, supervision, validation, visualization, writing – original draft, writing – review and editing. **Sangharsha Thapa:** conceptualization, formal analysis, methodology, resources, supervision, validation, writing – original draft, writing – review and editing. **Mustafa Abdulmahdi:** conceptualization, formal analysis, methodology, supervision, writing – original draft, writing – review and editing.

## Disclosure

This article has not been submitted to other publications or presented at conferences or meetings.

## Ethics Statement

Our institution does not require ethical approval to report individual cases or case series.

## Consent

Written informed consent was obtained from the patient to publish this report in accordance with the journal's patient consent policy.

## Conflicts of Interest

The authors declare no conflicts of interest.

## Data Availability

All data and results presented in this case report are open access and freely available to everyone. The full dataset supporting the findings of this study is included within the article and its supplementary materials. No restrictions apply to data access.
